# UC-USP collaborative exercise on photobiomodulation therapy in neurological orofacial disturbances

**DOI:** 10.4317/jced.56839

**Published:** 2020-07-01

**Authors:** Tiago Nunes, Catarina Caetano, Miguel Pimenta, José Saraiva, Salomão Rocha, Patrícia Freitas, José Figueiredo, Sónia-Alves Pereira, Ana Corte-Real

**Affiliations:** 1 Faculty of Medicine, University of Coimbra, Coimbra, Portugal; 2 Forensic Dentistry Laboratory, Faculty of Medicine, University of Coimbra, Coimbra, Portugal; 3 Special Laboratory of Lasers in Dentistry, School of Dentistry, University of São Paulo, São Paulo, Brazil

## Abstract

**Background:**

Neurosensory peripheral disorders are one of the most common risks associated with iatrogenic and/or post-traumatic injuries. It is often related to disability. Photobiomodulation therapy (PBMT) is a nonsurgical and safe procedure which can accelerate and improve the regeneration of injured biological tissue. This study aims to analyze the impact of PBMT, in the quality of life and impairment of individuals with orofacial neurological peripheral disturbance.

**Material and Methods:**

A retrospective analysis in the database of the dental traumatology clinic of the Hospital Centre of the University of Coimbra/Faculty of Medicine of the University of Coimbra was performed.5 out of 50 individuals were selected, according to the selection criteria. The neurosensory activity was assessed by a pinprick nociceptive test and the EQ-5D-5L self-report questionnaire was used to analyse the quality of life. The study was performed in two phases:1) inactive laser or placebo phase, for one month and 2) active laser or treatment phase. A diode low-level laser device (SIROLaserBlue;Sirona,Germany) was used, according to our protocol. A collaborative protocol in the PBMT influence in individuals with neurosensory peripheral disturbances was studied.

**Results:**

There was no improvement in the neurosensory activity nor in the quality of life, in the placebo phase. After the treatment phase, the EQ-5D-5L final results reported no problems in all of the five dimensions, except for anxiety/depression in individuals with long-standing neurosensory peripheral disturbances. The EQ-VAS scores increased in all the individuals.

**Conclusions:**

Our results supported the improvement of quality of life and impairment reduction in the individuals submitted to PBMT.

** Key words:**Low-level light therapy, peripheral nerve injuries, sensation disorders, quality of life, forensic medicine.

## Introduction

In the maxillofacial region, nerve injuries may occur as a result of trauma, neoplasms, infections and secondary to oral and/or maxillofacial surgical procedures (1), such as dental implant placement, orthognathic surgery, tooth extractions and surgical treatment of fractures (1-3). Nonsurgical procedures, including endodontic treatment and local anesthetics administration, can also cause nerve injuries (1,3).

Although many classifications for nerve injuries have been proposed, the two most widely used are those developed by Seddon and Sunderland (1,2,4). Seddon classifies the injuries by severity, with anatomic criteria, as neuropraxia, axonotmesis and neurotmesis (1,4), Sunderland focuses on the fascicular structure of the nerve and the remaining integrity following injury (1,4).

Nerve injuries are commonly associated with sensory disorders (5). Recently, the Association for the Study of Pain has categorized the most frequently used descriptive terms of neurosensory disorders as anesthesia, paresthesia or dysesthesia, each one with relative subcategories (6). Anesthesia refers to the total loss of feeling or sensation (6,7). Paresthesia is limited to an abnormal sensation that is not unpleasant (6,7). Dysesthesia represents any abnormal sensation that is unpleasant (6,7).

Although neurosensory recovery may be spontaneous, some injuries may be considered permanent, with sequelae ranging from a nonpainful minor loss of sensation and/or function to a major dysfunction (6,8). Neurosensory peripheral disorders often interfere with the individual’s ability to perform daily-life activities, eat comfortably, communicate clearly or manage oral secretions, resulting in disability (3,4,9).

In order to improve neurosensory recovery, surgical and acupuncture treatment alternatives may be available with variable outcomes and no consensus about the ideal approach (9).

Photobiomodulation therapy (PBMT) is a nonsurgical and safe procedure which can accelerate and improve the regeneration of injured biological tissue (10-13). PBMT is suggested to have an important role in inflammation, healing and neurological disturbances (14). PBMT consists on the application of a light that once absorbed promotes a photochemical biological effect exerting a chemical change (15). To produce any effect on a living biological system, the light must be absorbed by electronic bands belonging to some molecular photoacceptors, or chromophores, which can be seen in hemoglobin, flavoproteins, cytochrome c oxidase and myoglobin (15). The initial effects of light are thought to take place in the mitochondria and lead to biochemical and cellular changes such as increased cell proliferation and migration (particularly by fibroblasts), modulation in levels of cytokines, growth factors and inflammatory mediators and increased tissue oxygenation (14,16). This technique is referred to as Low-Level since the optimum dose of light delivered is low and not comparable to other forms of laser procedures used for cutting, ablation and thermal tissue coagulation (14,16). Lower or higher doses than the optimum value will have respectively, a minor therapeutic outcome or even a negative outcome (14,16).

This study is carried in cooperation with the Special Laboratory of Lasers in Dentistry, School of Dentistry - University of São Paulo, as a collaborative exercise. It aims to analyze the impact Low-Level Laser Therapy, in the quality of life and impairment, of individuals with orofacial neurological disturbance.

## Material and Methods

-Selection Strategy

A retrospective analysis in the database of the dental traumatology clinic of the Hospital Centre of the University of Coimbra / Faculty of Medicine of the University of Coimbra was performed. The individuals were selected, by the research team, according to the inclusion and exclusion criteria. Inclusion criteria were: age between 18 and 65 years; evaluation of orofacial civil damage; traumatic or iatrogenic etiology; orofacial impairment value, with medico-legal evaluation, secondary to functional and sensitive sequelae of the neurological system at March 2019. Exclusion criteria were the presence of: neurosensory disturbances before the procedures, concomitant therapy or medication, oncological diseases and incompatible area of residence.

-Clinical Evaluation of Peripheral Nerve Injury, Quality of Life and Impairment Assessment.

The research team carefully informed the individuals about the objectives of the study and asked them to agree to participate by signing an informed consent.

It was performed a self-report questionnaire, considering: the area affected, the assumption of traumatic or technical procedure cause, the chronological evolution and characterization of the symptoms, the consequences of the injury in the social, professional and daily life.

Each patient was asked to complete a quality of life questionnaire, EQ-5D-5L, before and after the treatment. The questionnaire consists of two parts: the EQ-5D-5L descriptive system and the EQ Visual Analogue scale (EQ-VAS). The descriptive system contains five dimensions (mobility, self-care, usual activities, pain/discomfort, anxiety/depression). Each dimension has 5 levels of severity (1-no problems; 2-slight problems; 3-moderate problems; 4-severe problems; 5-extreme problems). The respondent is asked to indicate his/her health state by ticking the box matching the most appropriate statement in each of the 5 dimensions. The EQ VAS records the respondent’s self-rated health on a visual analogue scale with endpoints labelled “the best health you can imagine” and “the worst health you can imagine.” (17). The English version of the questionnaire was used and a member of the research team would clarify any aspect the individuals would not comprehend.

-Objective examination 

The research team, three dental doctors with forensic/orthodontic/prosthodontic practice and one pre graduated student, proceeded with the individual/objective examination.

The neurosensory peripheral disturbance was assessed by a pinprick nociceptive test using a 30-gauge disposable needle to determine the patient’s sensibility response at the beginning of each treatment session. The patient was positioned comfortably with eyes closed, while one trained professional applied pressure with the tip of the needle and recorded the patient’s perception according to the response in a contralateral or nearest unaffected point as: (-) Total loss of sensation; (+) Partial loss of sensation; (#) Normal sensation (equal to control). Perception record and mapping were done by taking notes on a standardized drawing on a printed examination form. Additionally, a dermatograph pencil was used for mapping the affected area on the patient’s face and photographs were taken.

-PBMT Protocol

To address the research purpose, the study was performed in two phases: 1) inactive laser or placebo phase, for one month; 2) active laser or treatment phase and a follow-up visit.

A diode low-level laser device (SIROLaser Blue; Sirona, Bensheim, Germany) was used to irradiate the whole area enervated by the affected nerve twice a week. The laser device was set to deliver 100mW continuous wave at 660nm. The irradiation was performed in contact with the skin or mucosa using a light guide (MultiTip 8mm) perpendicular to the area to be treated for 40s per point, 4J per point, with a distance of 1cm between each irradiation point. If extra-oral irradiation was required, asepsis was performed on the patient’s skin with a 70% alcohol swab, to prevent harmful interference with substances in the absorption of the laser light.

In the first phase, placebo laser, the same protocol was used but the laser was inactive. This phase lasted 4 weeks, for a total of 8 sessions. In the active laser or treatment phase, the number of treatment sessions was determined by the patient’s clinical improvement, until total recovery was achieved. 1 month after the treatment phase was completed, a follow-up visit was scheduled.

## Results

**Figure 1:**
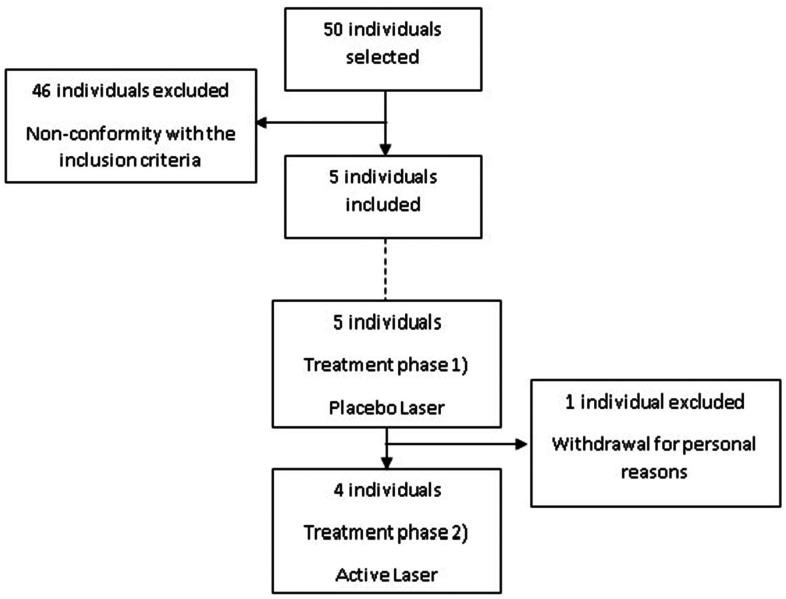
Flow diagram of patient selection.

A total of 50 individuals were enrolled in this study. 46 individuals were excluded for not meeting the inclusion criteria, ultimately providing 5 individuals for data analysis. During the treatment phase 1), 1 patient withdrawn from the study for personal reasons. The flow diagram of patient selection is shown in Figure 1.

All the included subjects were female with a mean age of 41 years old. The frequency of patient-reported daily-life activities in the initial examination is presented in Table 1. After the PBMT, treatment phase 2) was completed no disabilities were reported, ( Table 2, Table 3).

**Table 1:**
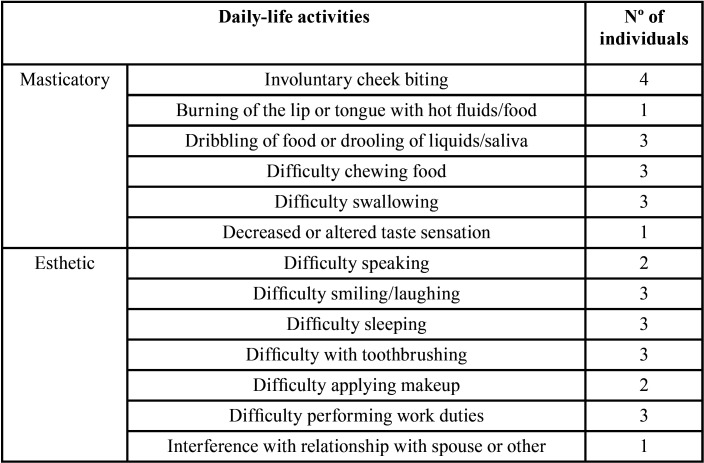
Frequency of patient-reported disabilities.

**Table 2:**
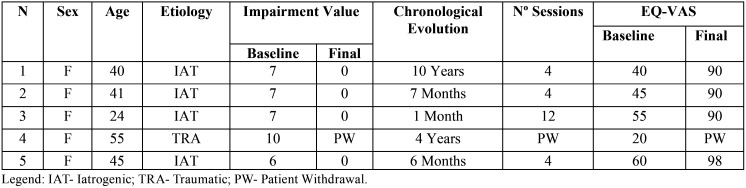
Demographic and neurosensory peripheral disturbance characteristics and EQ-VAS results.

**Table 3:**
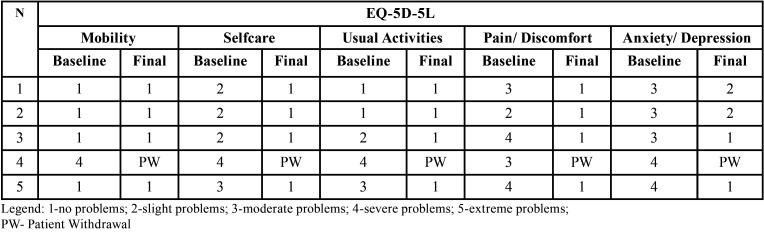
EQ-5D-5L descriptive system baseline and final reported levels of severity.

There was no change in the neurosensory recovery nor in the self-reported quality of life at the end of treatment phase 1), placebo laser.

## Discussion

A collaborative protocol in the PBMT influence in individuals with neurosensory peripheral disturbances was studied. A first evaluation was made based on the quality of life; a second evaluation was made based on the medico/legal evaluation of damage (impairment value).

The first randomized controlled clinical trial with PBMT on neurosensory deficits was reported in 1996. Until 2018, only 7 were disclosed (10,12,13,18-21). According to Chung *et al.* (22), PBMT dosimetry is highly complicated. Due to the large number of interrelated parameters (wavelength, irradiance, pulse structure, amongst others), there has not yet been a comprehensive study reporting and examining the effect of individually varying all those parameters, and it is unlikely there will ever be (22). The World Association for Laser Therapy (WALT) dosage guidelines for PBMT do not provide dose recommendations for neurosensory peripheral disturbances. The choice of parameters is often depended on the practitioner’s personal preference or experience rather than on a consensus statement by an authoritative body (22). None of the 7 studies (10,12,13,18-21) used a standardized protocol.

In this study, the laser device was set to deliver 100mW continuous wave at a wavelength of 660nm. The irradiation was performed in contact with the skin or mucosa using a light guide (MultiTip 8mm) perpendicular to the area to be treated for 40s an 4J per point, 1cm between each irradiation point.

The use of PBMT in animals and patients almost exclusively utilizes red and near-infrared light (600-1100nm) (15,16). Gigo-Benato *et al.* (23) demonstrated 660nm PBMT with low (10J/cm2) or moderate (60J/cm2) energy densities is able to accelerate neuromuscular recovery after nerve crush injury in rats.

The treatment sessions were performed twice a week so a cumulative effect of PBMT could be achieved. PBMT generally requires at least two treatment sessions a week for several weeks to achieve clinical significance, with the exception of some early treatment of acute injuries (22). A retrospective study of 125 clinical cases reported positive results in individuals given treatment twice a week (11).

Since each irradiated point will cover about 1-2cm3 of tissue, the whole area enervated by the injured nerve was irradiated with a distance of 1cm between each irradiation point to assure that a sufficient number of points are selected to cover the entire injury. The light guide, MultiTip 8mm, was used to cover a wider area on each irradiation point and it was used in contact with the tissue surface to reduce light reflection and improve light absorption.

The comparison between the studies’ results was hampered by the methodological non-standardization, explicitly in the clinical neurosensory disturbance evaluation. All of the studies (10,12,13,18-21) included objective testing, however with different methodologies. Only two studies (12,21) included a subjective assessment, which was a self-completed VAS scale in both cases. Our evaluation includes both an objective and subjective assessment. The objective assessment consisted in a pinprick nociceptive test, to determine the extent of the nerve injury and to monitor the neurosensory evolution. This method allows a simple measurement of response to static light touch and painful stimuli (24) and is also likely to detect a persistent sensory abnormality (25). Unlike any other study, the subjective assessment in our study focused on the quality of life evaluation.

To prepare the quality of life evaluation, an initial examination and questioning about the neurosensory peripheral disturbance, based on an article by Meyer *et al.* (24), was performed. Its purpose was to determine whether a sensory disturbance existed, what caused it, the orofacial functional impairment on the patient’s daily life and to ascertain whether PBMT was indicated. The EQ-5D-5L quality of life questionnaire, which also includes a VAS scale, is a standardized measure of health status, applicable to a wide range of injury conditions and treatments (17). It provides a simple descriptive profile that can be used in the clinical and health-economics evaluation of healthcare as well as in population health surveys (17). It was used in this study to record the individuals’ health profile and self-rated health status and to evaluate the individuals’ quality of life recovery. The EQ-5D-5L (scale with 5 levels of answer) was used instead of the EQ-5D-3L (scale with 3 levels of answer), since it could significantly increase reliability and sensitivity (discriminatory power) while maintaining feasibility and potentially reducing ceiling effects.

Our results support the improvement of quality of life (baseline mean value= 50; final mean value= 92). The results from the quality of life questionnaire showed a clear improvement both in the EQ-5D-5L descriptive system and the EQ-VAS. In the EQ-5D-5L descriptive system the final severity scores reported no problems in all of the five dimensions, with the exception of Anxiety/Depression in individuals with long-standing neurosensory peripheral disturbances. In those cases, there was still an improvement in the Anxiety/Depression dimension to slight level of problem, potentially a post-traumatic psychological sequela, which can pose as a bias of the study. The self-perceived health score, from the EQ-VAS, improved in all the individuals. In the long-standing cases, baseline scores were minor and the variances between the baseline and final scores were major, although the final scores were similar in all the cases.

Our results support the impairment reduction of all individuals submitted to Low-Level Laser Therapy. This item was never evaluated in any past study. The normal evolution of a neurological injury ends in its total recovery, that is, the cure. When this does not happen, the lesion becomes a sequela and may be associated with anatomical and/or functional, as well as psycho-social disability. In the context of orofacial neurological sequelae, its medico-legal/forensic valuation (impairment value) corresponds to the affectation of masticatory activity, speech and phonation. The professional technical/medical performance may allow the improvement of the clinical scenario and will tend to promote the improvement of the bio-psycho-functional capability of the individual.

Limitations of our study include small sample size, shortness of follow-up and patient dependent test responses. The difficulty of achieving a sample with the same clinical status poses a study bias.

## Conclusions

The results of the present study support a beneficial effect of Low-Level Laser Therapy on the improvement of quality of life as well as impairment reduction in individuals with traumatic and/or iatrogenic orofacial peripheral nerve injuries.

While the quality of life questionnaire evaluated the patient’s response, the medico-legal valuation provided an unbiased medical evaluation. The use of both approaches resulted in a complementary methodological relationship.

## References

[1] Steinberg MJ, Kelly PD (2015). Implant-related Nerve Injuries. Dent Clin North Am.

[2] Akal UK, Sayan NB, Aydoğan S, Yaman Z (2000). Evaluation of the neurosensory deficiencies of oral and maxillofacial region following surgery. Int J Oral Maxillofac Surg.

[3] Hillerup S (2008). Iatrogenic injury to the inferior alveolar nerve: etiology, signs and symptoms, and observations on recovery. Int J Oral Maxillofac Surg.

[4] Steed MB (2011). Peripheral Nerve Response to Injury. Atlas Oral Maxillofac Surg Clin North Am.

[5] Alhassani AA, AlGhamdi AST (2010). Inferior Alveolar Nerve Injury in Implant Dentistry: Diagnosis, Causes, Prevention, and Management. J Oral Implantol.

[6] Misch CE, Resnik R (2010). Mandibular nerve neurosensory impairment after dental implant surgery: Management and protocol. Implant Dent.

[7] Juodzbalys G, Wang H L, Sabalys G (2011). Injury of the Inferior Alveolar Nerve during Implant Placement: a Literature Review. J Oral Maxillofac Res.

[8] Ziccardi VB, Zuniga JR (2007). Nerve Injuries After Third Molar Removal. Oral Maxillofac Surg Clin North Am.

[9] Leung YY, Fung PPL, Cheung LK (2012). Treatment modalities of neurosensory deficit after lower third molar surgery: A systematic review. J Oral Maxillofac Surg.

[10] Gasperini G, de Siqueira ICR, Costa LR (2014). Lower-level laser therapy improves neurosensory disorders resulting from bilateral mandibular sagittal split osteotomy: a randomized crossover clinical trial. J Craniomaxillofac Surg.

[11] de Oliveira RF, de Andrade Salgado DMR, Trevelin LT, Lopes RM, da Cunha SRB, Aranha ACC (2015). Benefits of laser phototherapy on nerve repair. Lasers Med Sci.

[12] Mohajerani SH, Tabeie F, Bemanali M, Tabrizi R (2017). Effect of Low-Level Laser and Light-Emitting Diode on Inferior Alveolar Nerve Recovery After Sagittal Split Osteotomy of the Mandible: a Randomized Clinical Trial Study. J Craniofac Surg.

[13] Fuhrer-Valdivia A, Noguera-Pantoja A, Ramirez-Lobos V, Sole-Ventura P (2014). Low-level laser effect in patients with neurosensory impairment of mandibular nerve after sagittal split ramus osteotomy. Randomized clinical trial, controlled by placebo. Med Oral Patol Oral Cir Bucal.

[14] Hamblin MR, Demidova TN (2006). Mechanisms of low level light therapy.

[15] Huang YY, Chen ACH, Carroll JD, Hamblin MR (2009). Biphasic dose response in low level lightherapy. Dose-Response.

[16] Farivar S, Malekshahabi T, Shiari R (2014). Biological effects of low level laser therapy. J lasers Med Sci.

[17] Van Reenen M, Janssen B (2005). EQ-5D-5L User Guide Basic information on how to use the EQ-5D-5L instrument.

[18] Khullar SM, Emami B, Westermark A, Haanaes HR (1996). Effect of low-level laser treatment on neurosensory deficits subsequent to sagittal split ramus osteotomy. Oral Surg Oral Med Oral Pathol Oral Radiol Endod.

[19] Khullar SM, Brodin P, Barkvoll P, Haanaes HR (1996). Preliminary study of low-level laser for treatment of long-standing sensory aberrations in the inferior alveolar nerve. J oral Maxillofac Surg.

[20] Eshghpour M, Shaban B, Ahrari F, Erfanian M, Shadkam E (2017). Is Low-Level Laser Therapy Effective for Treatment of Neurosensory Deficits Arising From Sagittal Split Ramus Osteotomy?. J oral Maxillofac Surg.

[21] Miloro M, Criddle TR (2018). Does Low-Level Laser Therapy Affect Recovery of Lingual and Inferior Alveolar Nerve Injuries?. J Oral Maxillofac Surg.

[22] Chung H, Dai T, Sharma SK, Huang YY, Carroll JD, Hamblin MR (2012). The nuts and bolts of low-level laser (light) therapy. Ann Biomed Eng.

[23] Gigo-Benato D, Russo TL, Tanaka EH, Assis L, Salvini TF, Parizotto NA (2010). Effects of 660 and 780 nm low-level laser therapy on neuromuscular recovery after crush injury in rat sciatic nerve. Lasers Surg Med.

[24] Meyer RA, Bagheri SC (2011). Clinical Evaluation of Peripheral Trigeminal Nerve Injuries. Atlas Oral Maxillofac Surg Clin.

[25] Robinson PP (1988). Observations on the recovery of sensation following inferior alveolar nerve injuries. Br J Oral Maxillofac Surg.

